# Craniofacial trauma and double epidural hematomas from horse training^☆^

**DOI:** 10.1016/j.ijscr.2013.10.011

**Published:** 2013-11-07

**Authors:** Aaron D. Baugh, Reginald F. Baugh, Joseph N. Atallah, Daniel Gaudin, Mallory Williams

**Affiliations:** a University of North Carolina, Chapel Hill, United States; b Division of Otolaryngology, University of Toledo College of Medicine, United States; c Department of Anesthesiology, University of Toledo College of Medicine, United States; d Division of Neurosurgery, Department of Surgery, University of Toledo College of Medicine, United States; e Division of Acute Care Surgery, University of Toledo College of Medicine, 3000 Arlington Avenue, Mailstop 1095, Toledo, OH 43623, United States

**Keywords:** Double epidural hematoma, Equestrian, Supraorbital neuralgia, Traumatic cranial nerve, Palsy, Arytenoid subluxation, Temporal bone fracture

## Abstract

**INTRODUCTION:**

A case of complex poly-trauma requiring multi-service management of rare, diagnoses is reviewed.

**PRESENTATION OF CASE:**

A healthy 20 year old female suffered double epidural hematoma, base of, skull fracture, traumatic cranial nerve X palsy, benign positional paroxysmal vertigo and supraorbital, neuralgia following equestrian injury.

**DISCUSSION:**

Epidemiology, differential diagnosis, and principles of management for each condition, are reviewed.

**CONCLUSION:**

Coordinated trauma care is well suited to address the complex poly trauma following, equestrian injury.

## 1 Introduction

Horseback riding is naively considered by many to be a safe, gentle sport with few risks. The reality is that it can be more dangerous than motorcycle riding, skiing, automobile racing, or football. Most riding injuries occur when the rider is either thrown or falls from the horse. Experience is no protection, as experienced riders account for one third of all injuries.^1^ Relative to other activities, participants are subjected to high forces with comparatively little protective gear, creating the potential for severe injury. Consistent with this scenario, chronic physical impairment follows equestrian injury more than half the time.^2^ One such rare injury is double epidural hematomas (DEDH) with an overall incidence of 2–10% of all epidural hematomas.^3^ This injury has a mortality rate of greater than 30%.^4^ Our case report describes a patient who suffers from DEDH combined with other major CNS and maxillofacial trauma secondary to being thrown from a horse and survives. We will focus on the presentation, diagnosis, and management of these rare injuries.

## 2 Presentation of case

A healthy 20 year old female ranch hand was thrown from an untamed horse she was attempting to break. She was not wearing any protective head gear at the time of her injury. Prior to her injury, she had 3 years experiences as a ranch hand and frequent interaction with horses in both vocational and recreational contexts since early childhood. She was found by co-workers, who witnessed emesis and lateral nystagmus. No medical professionals were available on-site. Emergency medical services assessed her as convulsive in all extremities with an overall Glasgow Coma Scale of 10. The patient was intubated in the field. During transit to the trauma center via Life Flight, the patient self-extubated. She was re-intubated before arrival. The patient reached the ER hemodynamically stable, intubated, and sedated.

**Fig. 1:**
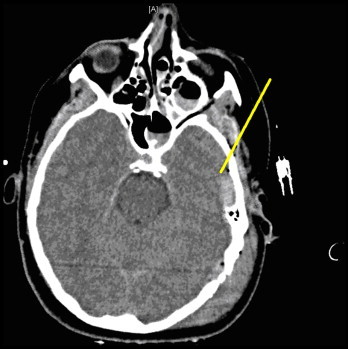
Left temporal bone fracture, pneumocephalus and left-sided epidural hematoma.

**Fig. 2:**
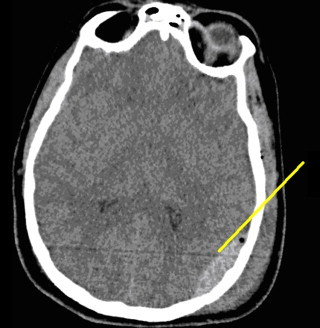
Left parietal skull fracture with second of two left-sided epidural hematomas, pneumocephalus and overlying scalp contusion.

The patient reached the ER hemodynamically stable, intubated, and sedated. Computed tomography (CT) of the head demonstrated epidural hematomas in both the left temporal and parietal areas and fractures of the zygoma, posterior skull, and temporal bones (Figs. 1 and 2). A Camino ventriculostomy drain was placed for initial intracranial pressure (ICP) monitoring.

An emergent craniotomy was required for progression of the epidural hematomas and increasing ICP measurement. Mannitol therapy and intra-operative drain placement were used to ICP control through the third hospital day.

Severe headaches began on the fourth hospital day. She described her pain intensity as 10/10 in a frontal distribution that radiated to the back of the head. The patient was found to have exquisite tenderness and paresthesias in the distribution of the supra-orbital nerves suggesting supra-orbital neuralgia. Bilateral supra-orbital nerve block was diagnostic. Treatment with local anesthesia and steroids was successful for long-term pain control.

Persistent otalgia, significant hoarseness, aspiration, severe dysphagia to liquids and multiple episodes of transient vertigo of <30 s duration, triggered by rapid changes in head position were noted. On exam, the left bony canal and tympanic membrane were clearly distorted. Weber lateralized to the left and left-sided Rinne test showed bone conduction greater than air conduction. The left temporomandibular joint (TMJ) was tender to palpation. Gag reflex was diminished on the left. Flexible laryngoscopy revealed an immobile left true vocal cord. She failed a bedside swallow test. The remainder of the head and neck exam was within normal limits.

Otalgia was attributable to the injured tympanic membrane and the TMJ fracture. A modified barium swallow demonstrated aspiration of thin liquids. A thick soft diet was used to prevent further aspiration events. Visualized injuries during the left otoscopic exam and accompanying hearing loss were suggestive of a possible injury to the ossicular chain. The patient's vertigo was suggestive of traumatic benign positional paroxysmal vertigo.

At 1 month follow-up, the patient's hoarseness had significantly improved but some harshness remained. On video stroboscopy, a moderate sized acute edematous polyp on the left true vocal cord was identified. Vocal cord motion was now present bilaterally. The patient reported only occasional choking with rapid swallowing of liquids. The left gag reflex had also returned to normal.

**Fig. 3:**
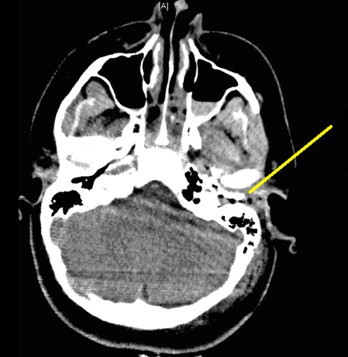
Fracture through the external auditory canal anterior and posterior walls.

While audiologic improvement at 2000 Hz was noted, left-sided high frequency hearing loss remained. The external auditory canal was grossly abnormal with the inferior portion of the tympanic membrane obscured by an anterior bony canal mass. Computed tomography (CT) findings from the day of the trauma included post-traumatic changes in the middle ear, and bone fragments in the external auditory canal (Fig. 3).

## 3 Discussion

Our patient suffered a very rare constellation of CNS and maxillofacial injuries. Our discussion includes the following injuries: double epidural hematomas, hearing loss vertigo, supraorbital neuralgia, and dysphonia.

### 3.1 Double epidural hematomas (DEDH)

The etiology of EDH is a laceration of the middle meningeal artery. The need for craniotomy is determined by either the volume of hematoma (>30 cm^3^) on CT or physical examination.^5^ A physical examination demonstrating neurological deterioration, papillary signs, or impending herniation are criteria for craniotomy. The mortality is from the underlying brain injury, and not the EDH itself.

DEDH is an extremely lethal and rare diagnosis, infrequently described in the medical literature. The mortality for DEDH exceeds 30%.^4^ Unlike the classic epidural hematoma, DEDH are most often venous in origin.^6^ Simultaneous evacuation is the preferred management. The frontal lobe is the most common location. The reported incidence in most series is 2–10% of EDH, with the unilateral variant comprising only 8.6% of that subgroup.^4^ Both variants is associated with higher mortality, lower presentation GCS, lower likelihood of a lucid interval, and greater tendency for clinical deterioration.^4,6^ Our patient had a unilateral DEDH and despite having all of these characteristic presentations including clinical deterioration, she survived. Our view is that rapid transport to a Level I trauma center played a significant role in this patients survival.

### 3.2 Hearing loss

Hearing loss is common after temporal bone fractures. Conductive hearing loss is common with longitudinal fractures because of their potential transverse of the middle ear space. Hemotympanium, ossicular disruptions, and tympanic membrane perforations are commonly observed, but resolve in 3–4 weeks as the middle ear clears. Disruption of the otic capsule results in severe to profound neurosensory hearing loss and is more common with transverse fractures. An audiologic evaluation is warranted in the immediate post-injury state in suspected facial nerve injury, nystagmus, or persistent CSF leak. Our patient experience minimal recovery in the human speech range of hearing but demonstrated persistent deficits in the high frequency range at 1 month follow up.

### 3.3 Vertigo

Trauma is the most common etiology for benign paroxysmal positional vertigo. Otoconia in the vestibular canals disrupt the normal hair cell function, sending false indicators of motion. The resulting vertigo is usually triggered by turning the head in certain directions, and lasts only a few seconds. When stimulated, the nystagmus is fixed, horizontal or horizonto-rotatory lasts typically seconds and fatigues. Though most respond to a canalith re-positioning maneuver, although traumatic BPPV may be refractory to treatment.^7^

The intensity of the vertigo will improve in about a week regardless of etiology, with complete resolution expected for traumatic BPPV. After a year, stability of symptoms is reached by most patients as most compensation has occurred by then. Older patients are more likely to have incomplete resolution of symptoms. Vestibular rehabilitation is often useful. Our patient had resolution of her traumatic BPPV in less than 1 month with vestibular rehabilitation.

### 3.4 Supraorbital neuralgia

Cardinal features of the supraorbital neuralgia are a positive Tinel's sign at the supra-orbital notch, and pain and hypoesthesia in the nerve's distribution.^8^ Supraorbital nerve block is the preferred treatment. It is highly efficacious by expert consensus, but clinical trials have not formally investigated this question.^9^ The differential diagnosis includes other headache syndromes and neuralgias. Sudden unilateral neuralgiform headache with conjunctival tearing and injection (SUNCT) syndrome, while highly refractory to medical treatment, is male-predominant, unilateral, and more commonly refractory to treatment by nerve blockade.^10^ Migraine should demonstrate more associated photophobia, phonophobia, and nausea, while cluster headache is more characterized by autonomic involvement. Other neuralgias should show point tenderness in their respective anatomical distributions.

### 3.5 Dysphonia

Vocal cord dysfunction is both a significant functional impairment and contributor to dysphagia. The persistence of either finding merits evaluation of vocal cord function. Breathy dypshonia is particularly concerning. In this case, possible causes of dysphonia included direct injury to the laryngeal innervation or subluxation of the arytenoid. Video stroboscopy can help visualize and differentiate these pathologies.

Arytenoid subluxation is injury to the cricoarytenoid joint. While reported after traumatic extubation like this case's, its rarity and irreproducible mechanism lead many to question the existence of the diagnosis.^11^ Basilar skull fractures can result in injuries to the vagus, accessory or hypoglossal nerves as they exit the skull, although isolated injury to a single nerve is rare.^12–15^ Case history was most consistent with this etiology. Lower cranial nerve palsy in the setting of a head injury should prompt careful examination of the skull base to exclude significant fractures. The presentation of the palsy maybe delayed in its presentation so a careful reassessment of the patient may be warranted. Treatment is conservative acutely. Longer term rehabilitation efforts focus on vocal function.

## 4 Conclusion

DEDH is a severe CNS injury with distinct presentation and high mortality. Relative to single epidural hematomas, it is characterized by lower GCS at presentation, less likelihood of a lucid interval, and neurological deterioration. DEDH in association with other CNS and maxillofacial injuries may be survivable with appropriate trauma care. However, morbidity remains high.

## Conflict of interest

The authors of this work have no conflicts of interest to declare.

## Funding

None.

## Ethical approval

Written informed consent was obtained from the patient for publication of this case report and case series and accompanying images.

## Authors contribution

All authors contributed to data analysis and writing those sections of the manuscript that pertained to their particular area of specialty expertise in this case of polytrauma management.
